# Factors associated with and patterns of alcohol intake in late survivorship for breast cancer survivors

**DOI:** 10.21203/rs.3.rs-8492613/v1

**Published:** 2026-01-29

**Authors:** Sanjna Rajput, Robert A. Vierkant, Kayleigh N. Olson, Nicole L. Larson, Daniela L. Stan, Dawn M. Mussallem, Stacy D. D’Andre, Fergus J. Couch, Janet E. Olson, Ciara C. O’Sullivan, Kathryn J. Ruddy

**Affiliations:** Mayo Clinic

## Abstract

**Purpose:**

To evaluate alcohol intake trends and identify demographic, clinical, lifestyle and socioeconomic factors associated with alcohol consumption in late survivorship among breast cancer survivors.

**Methods:**

Individuals diagnosed with stage 0–3 breast cancer enrolled in the Mayo Clinic Breast Disease registry between 2014 to 2022 reported their average weekly alcohol intake at baseline (time of diagnosis) and at approximately four years post-diagnosis. Alcohol intake was divided into four categories and cross-sectional associations with demographic, clinical, and lifestyle factors were examined using Monte Carlo-based Fisher exact tests and multivariable multinomial logistic regression. Changes in alcohol consumption from baseline to Year 4 were evaluated using Bowker’s test of symmetry and multinomial models.

**Results:**

Among 719 participants, alcohol intake 4 years post diagnosis closely resembled baseline patterns, with 30.2% of patients reporting no alcohol use and 48.8% of patients consuming 1–4 drinks per week. Younger age and current smoking status were strongly associated with higher intake at Year 4. Exercise and better physical health were associated with higher alcohol intake in univariable models, however not in adjusted models. From time of diagnosis to Year 4, 15.6% of patients decreased their alcohol intake, 10.2% increased alcohol intake, and 74.3% reported no change. Higher levels of mild intensity exercise were associated with an elevation in alcohol intake over time.

**Conclusion:**

Alcohol consumption remained stable in late survivorship, in contrast to the initial decline in alcohol use during early survivorship seen in this cohort. Younger age and smoking were key associations with higher alcohol intake.

## INTRODUCTION

Breast cancer (BC) is the most common cancer diagnosis among women in the United States,^1^ and in 2020, the worldwide BC incidence surpassed lung cancer for the first time.^2^ In recent decades, the overall incidence rates of BC in females have increased, specifically by 1% annually since 2012.^1^ With advances in systemic and local treatments as well as earlier detection through improved screening, death rates due to BC have decreased 44% since 1989, preventing over 500,000 estimated deaths.^1^ Given high rates of survival, with the five-year survival rate for BC exceeding 90%, it is anticipated that an ever-increasing population will be living with, or be in remission from BC.^1^ Thus, evaluating and addressing factors which negatively influence BC outcomes is of the utmost importance.

Alcohol is a known carcinogen and has been linked to an increased BC incidence.^3-7^ Patients with a history of certain cancers, including BC, significantly benefit from healthy lifestyle choices.^8^ Approximately 30% of BC cases can be attributed to modifiable lifestyle factors, including alcohol intake.^6^ Recurrence and mortality have been found to be impacted by both pre- and post-diagnosis alcohol consumption levels, with studies suggesting that higher alcohol consumption is associated with an increased risk of overall mortality, and postdiagnosis alcohol intake is positively associated with breast cancer recurrence.^4^ Some studies also suggest a higher risk of recurrence in postmenopausal but not premenopausal women who regularly consume ≥ 6 grams of alcohol daily post-diagnosis (HR, 1.19; 95% CI, 1.01–1.40).^9^

Most of the available evidence has focused on the relationship between alcohol intake and BC incidence, and the results are relatively consistent.^10^ Less has been published on post-diagnosis alcohol intake and the risk of BC recurrence. Additionally, there are little or no studies assessing alcohol intake in late BC survivorship (often defined as the period from 2 to 5 years post-diagnosis).^11-13^ Multiple studies show that, following a BC diagnosis, patients are likely to alter their dietary habits and alcohol intake (i.e., eating a more balanced diet and/or reducing alcohol intake).^14-15^ While cancer survivorship guidelines suggest limiting alcohol intake, if not avoiding it altogether,^16^ some cancer survivors continue to consume alcohol, and may even increase their intake post BC diagnosis. Studies suggest roughly 75% of cancer survivors drink alcohol post diagnosis, with many partaking in risky consumption patterns (consuming > 2 drinks per day, binge drinking i.e. consuming ≥ 6 drinks on a single occasion, hazardous drinking i.e. AUDIT-C scores ≥ 3).^14, 17-18^ Many studies do not monitor long-term alcohol intake in BC survivors, which is critical as these patients are now living longer than ever before.

Our group previously studied demographic, lifestyle, and clinical characteristics associated with changes in alcohol intake in a cohort of BC survivors in early survivorship, i.e., in the year following BC diagnosis.^19^ However, alcohol use is not static across survivorship; it is shaped by evolving clinical, psychological, and social factors. Our current study therefore aims to assess changes in and associations with alcohol intake in the same cohort of BC survivors later in survivorship, i.e. four years following BC diagnosis.

## METHODS

Individuals aged 18 or older who were diagnosed with BC within the past year and had at least one clinical visit at the Mayo Clinic in Rochester, Minnesota, were prospectively enrolled in the Mayo Clinic Breast Disease Registry (MCBDR) upon providing informed consent. Participants diagnosed with BC between 2014 and 2022 were asked to provide information regarding their alcohol intake on the initial (baseline) survey and again at approximately four years after diagnosis.

Individuals who completed the alcohol intake question on both the baseline and four-year follow-up surveys were included in the analysis. Exclusion criteria included a diagnosis of stage IV BC, a documented BC recurrence prior to the completion of the Year 4 survey, or a baseline survey submitted more than one year after the initial diagnosis.

The baseline and Year 4 surveys assessed self-reported average number of alcoholic drinks consumed per week over the past 10 years, defining a standard drink as 5 ounces of wine, 12 ounces of beer, or 1 ounce of liquor. Reported alcohol use was categorized into four weekly intake levels: none, low (1–4 drinks), moderate (5–14 drinks), and high (15 or more drinks).

Participants provided clinical data and additional demographic information regarding their education level, the presence or absence of financial strain (e.g., difficulty paying bills or limited disposable income), their smoking behavior (never, former, current), exercise levels, menopausal status, and PROMIS-10 health scores. For exercise levels, participants reported the number of minutes per day and days per week of engagement in mild, moderate and strenuous exercise. Mild-intensity activity was defined as requiring “minimal effort”, moderate-intensity activity as “not exhausting” and strenuous activity as “heart beats rapidly”. The PROMIS Global-10 instrument consists of 10 items evaluating overall health, well-being, and distress, yielding scores for global physical health (GPH) and global mental health (GMH).^20^ These scores are presented as T-scores derived from raw totals, where a T-score of 50 represents the average of the U.S. general population, with a standard deviation of 10. Higher T-scores indicate better physical or mental health. T-scores were categorized based on approximate quartiles amongst all participants returning baseline questionnaires. Data was abstracted by trained nurses from electronic medical records—including patient race, gender, age at diagnosis, cancer stage and features, BRCA mutation status, and post-diagnosis treatments (such as surgery, radiation, chemotherapy, and/or endocrine therapy)—were incorporated into the analysis.

## Statistical methods

Data were summarized using frequencies and percents for categorical variables and means and standard deviations for continuous variables. Associations of participant attributes with return of the Year 4 questionnaire, among all patients returning the baseline questionnaire, were assessed using chisquare tests.

Among participants returning the Year 4 questionnaire, univariable cross-sectional associations of Year 4 alcohol intake with demographic and clinical characteristics were carried out using Monte Carlo-based Fisher exact tests. In brief, for a given comparison, a cross-tabulated table was created using the observed data and the Fisher test statistic was calculated. Following this, 10,000 random samples of cross-tabulated tables were generated, conditional on the same total sample size, column totals, and row totals as the observed table. The Monte Carlo p-value was then determined as the proportion of the resulting 10,000 tables that yielded a test statistic at least as large as the one observed. We chose Monte Carlo-based tests rather than traditional Fisher exact tests because the number of permutations needed to calculate the exact tests was extremely large and memory intensive. Following these univariable associations, we fit a nominal, multivariable, multi-categorical logistic regression model to determine variables independently associated with Year 4 alcohol intake. Alcohol intake was fit as the outcome, and all variables univariably associated with alcohol intake (p < 0.05) were included in the model, using a multinomial distribution and a generalized logit (glogit) link function. Because multinomial logistic regression odds ratios can be challenging to interpret, we instead presented estimated marginal percents of participants falling within a given alcohol intake category for each level of each characteristic of interest, similar to the calculation of least squares means for continuous outcome variables. First, predicted probabilities of alcohol intake were calculated for each combination of all variables in the model. Then, for a given level of a given characteristic of interest, the mean of the predicted probabilities of alcohol intake for that level were determined by averaging all predicted probabilities for that level across all combinations of the other variables in the model. These marginal predicted probabilities were multiplied by 100 to arrive at predicted percents.

Change in alcohol intake from baseline to Year 4 was assessed using Bowker’s test of symmetry. Following this, we examined univariable associations of changes in alcohol intake from baseline to Year 4 with characteristics of interest, again using Monte Carlo-based Fisher exact tests. For these associations, data were subset to participants with baseline alcohol intake of 1–4 or 5–14 drinks per week, since participants at the lower extreme of alcohol intake (< 1 drink per week) could not decrease alcohol consumption at Year 4 and those at the higher extreme (15 + drinks per week) could not increase alcohol consumption at Year 4. Participants were grouped into three change categories based on change in alcohol intake from baseline: decreased alcohol intake at Year 4, no change in alcohol intake at Year 4, increase in alcohol intake at Year 4. Multivariable analyses again included all variables found to be univariably significant (p < 0.05), and resulting marginal percents calculated, similar to the Year 4 cross-sectional analyses.

All statistical tests were two-sided, and all analyses were carried out using the SAS system (SAS Institute, Inc., Cary, NC).

## RESULTS

A total of 2230 patients with stage 0-3 BC returned a baseline questionnaire. Of these, we excluded 1502 who either did not return the Year 4 questionnaire or provided insufficient alcohol intake data at baseline or at Year 4, 2 whose BC recurred prior to the Year 4 questionnaire, and 7 who returned their baseline survey more than one year after their original diagnosis, resulting in a final cohort of 719 patients. Associations of participant characteristics with return of the Cohort 4 questionnaire, amongst all 2230 patients, are provided in Supplemental Table 1. Patients who were white, premenopausal, with Stage 0-2 disease, who did not undergo chemotherapy or radiotherapy, and who had higher PROMIS-10 mental and physical health scores were more likely to return the Year 4 questionnaire than their demographic and clinical counterparts.

Cohort characteristics are provided in Table 1. The mean age at BC diagnosis was 58.6 years (SD = 12.0). Ninety-nine percent of participants were female and 97% were of White race. Most participants were post-menopausal (74%). Approximately two-thirds of participants were never smokers (67%). More than half held a bachelor’s or graduate-level degree. One quarter of participants reported financial difficulty and 27% reported not engaging in at least mild exercise per week. Nearly half of participants had stage 1 BC (49%), and the majority of tumors were ER positive (85%), PR positive (77%) and HER2 negative (88%). Slightly more than half of the participants (51%) underwent lumpectomies, 66% received endocrine therapy, 27% received chemotherapy and 52% received radiotherapy.

## Cross-sectional associations of alcohol intake at Year 4 with participant characteristics

**Figure 1:**
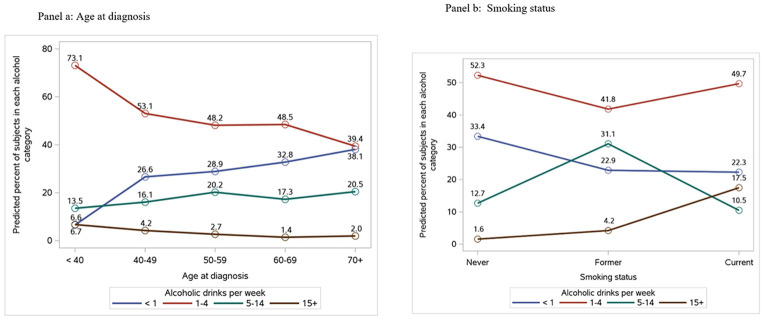
Effect plots of the variables that were significant in the multivariable models of associations of demographic and clinical characteristics with alcohol intake at Year 4

Of the 719 participants, 217 (30%) reported little or no alcohol intake, 351 (49%) consumed 1-4 drinks per week, 134 (19%) consumed 5-14 drinks per week and 17 (2%) reported consuming 15 or more drinks per week. Univariable associations of alcohol intake at year 4 with participants characteristics are provided in Table 1. Lower alcohol consumption was associated with older age at diagnosis, such that 38.5% of participants aged 70 and older reported no alcohol intake compared to 6.4% of participants < 40 (p<0.001) (Table 1, Figure 1). Thirteen percent of current smokers reported consuming 15 or more alcoholic drinks per week compared to 3.7% for former smokers and 1.5% for never smokers (p<0.001). Alcohol intake was lower in participants reporting zero minutes of moderate exercise per week (p=0.001) and in participants reporting zero minutes of mild exercise per week (p=0.005) compared to those reporting at least some exercise. Similarly, participants with lower PROMIS global physical health scores were more likely to abstain from alcohol compared to those with higher scores: 71% with scores < 46 abstained compared to 54% with scores of 56 or greater (p<0.001). No significant associations were observed between alcohol intake and tumor-related or treatment characteristics (p>0.05 for each).

Age at diagnosis remained a significant correlate of alcohol consumption in multivariable analyses, in that 6.6% of participants less than age 40 reported no alcohol intake compared to 38.1% of participants 70 and older (p=0.022, Figure 1a, Supplemental Table 2). Smoking also remained significantly associated with alcohol consumption, such that 22% of current smokers abstained and 17.5% consumed 15 or more drinks per week compared to 33.4% and 1.6% for never smokers (p<0.001, Figure 1b, Supplemental Table 2). Exercise and physical health scores were no longer significantly associated with alcohol intake in multivariable analyses.

## Trend in alcohol intake from Baseline to Year 4

The proportion of participants falling into each alcohol intake category at Year 4 was not significantly different from that at baseline (Bowker test for homogeneity p=0.13): 27.7% reported no alcoholic drinks per week at baseline compared to 30.2% at Year 4, 48.3% reported 1-4 weekly drinks at baseline compared to 48.8% at Year 4, 21.0% reported 5-14 drinks per week at baseline compared to 18.6% at Year 4, and 3.1% reported 15+ drinks per week at baseline compared to 2.4% at Year 4 (Table 2). Of the 719 participants, 73 (10.2%) increased alcohol intake from baseline to Year 4, 112 (15.6%) decreased intake and 534 (74.3%) did not change their intake.

## Associations of change in alcohol intake from baseline to Year 4 with participant characteristics

**Figure 2:**
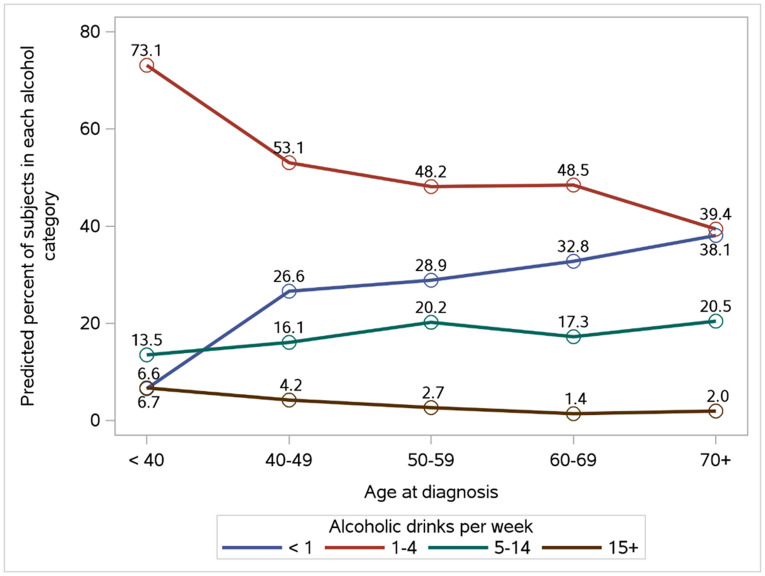
Effect plot of multivariable association of weekly minutes of mild exercise per week with change in alcohol intakefrom Baseline to Year 4

Univariable associations of change in alcohol use from baseline to Year 4 with demographic and clinical characteristics, among the 498 participants not at the lowest or highest levels of baseline alcohol use, are presented in Table 3. Increases in alcohol consumption were more common in participants reporting 60-119 (13.0%) and 120+ minutes of strenuous exercise per week (15.9%) compared to those reporting 0 (7.5%) or 1-59 minutes per week (6.9%, p=0.011). Conversely, decreases in alcohol consumption were more common in participants reporting 0 (21.0%) or 1-59 minutes of strenuous exercise per week (34.5%) compared to those reporting 120+ minutes per week (7.2%, p=0.011). Similarly, participants reporting 240 or more weekly minutes of mild exercise per week were more likely to increase their alcohol consumption (18.4%) compared to those with 0 (9.7%), 1-119 (7.0%) or 120-239 minutes per week (4.6%, p=0.012). A lower proportion of participants who received endocrine therapy decreased their alcohol consumption from baseline to Year 4 (16.8%) than those who did not receive therapy (25.6%, p=0.047). No other demographic or clinical characteristics were significantly associated with changes in alcohol consumption (p>0.05 for each). In multivariable analyses, the only attribute associated with change in alcohol consumption was weekly minutes of mild exercise per week (p=0.026, Figure 2, Supplemental Table 3). Results were similar to those seen in univariable analyses, in that participants reporting lower levels of exercise were more likely to decrease their alcohol consumption compared to those reporting higher levels, and those reporting 240 or minutes of mild exercise per week were more likely to increase consumption compared to those reporting lower exercise levels.

## DISCUSSION

In this prospective cohort study of BC survivors, we examined clinical, demographic, lifestyle, and socioeconomic factors associated with alcohol consumption four years after diagnosis, as well as changes in alcohol use from the time of diagnosis to late survivorship. Our group has previously found that in early survivorship (Year 1), participants were noted to overall reduce alcohol consumption compared to baseline, with the proportion of participants consuming <1 drink/week increasing from 28.9% at baseline to 60.2% at Year 1 and those drinking ≥1 drink/week declined from 71.2% at baseline to 39.8% at Year 1.^19^ Our new findings extend our prior research by evaluating behavior at a later point in the survivorship trajectory. This later timepoint captures a distinct and underexamined phase in survivorship, during which behavior patterns may stabilize or shift in response to long-term psychosocial adjustment, evolving health status, and changes in care intensity.

Consistent with prior studies in cancer populations, younger age at diagnosis was strongly associated with higher levels of alcohol intake in both univariable and multivariable cross-sectional analyses of alcohol use at Year 4. Studies have shown that cancer survivors <65 years of age are more likely to engage in current alcohol consumption, exceed moderate drinking and engage in binge drinking compared to those aged 65 years and older.^21-22^ These findings highlight the need for targeted interventions to address alcohol consumption and support sustainable behavioral change among younger cancer survivors, given the potential adverse effects on treatment outcomes and overall health.

Current smoking status remains a strong and consistent association with higher alcohol intake at Year 4 and across all timepoints including in our prior studies at baseline and Year 1. This is consistent with well-documented clustering of health risk behaviors. Continued smoking through several timepoints in survivorship may be influenced by comorbid psychological distress, anxiety and depression as well as insufficient cessation support.^23-24^ This dual-risk profile of combined alcohol and tobacco use has particular relevance for survivorship care, as combined use is associated with worse cancer outcomes such as recurrence and development of subsequent primary cancers as well as overall mortality.^25-26^ Identifying individuals with multiple behavioral risk factors highlights the need for increased psychologic support over time for affected patients and can help integrate behavioral counseling approaches more effectively.

In a multivariable model, survivors reporting higher levels of mild or strenuous exercise were found to be more likely to increase alcohol intake, while those with lower activity levels were more likely to reduce consumption. This finding mirrors the literature suggesting that some individuals may compensate for alcohol consumption through healthy behaviors such as exercise or may socialize more frequently in physically active settings that promote alcohol use.^27-29^

Treatment-related variables were not significantly associated with alcohol intake in cross-sectional models. This is a change from analyses at Year 1 revealing an association of chemotherapy with reduction in alcohol intake (which may have been due to chemotherapy-related taste-disturbances and nausea). This may reflect a decoupling of medical and behavioral trajectories as patients transition from active treatment into survivorship.

Our study also found that most BC survivors in our cohort (74.3%) reported a similar amount of alcohol consumption at baseline and at 4 years following diagnosis. Only 15.6% reported a decrease and 10.2% reported an increase in intake. This is a significant difference from the declines seen in alcohol use in this cohort one year after diagnosis. This suggests that the greatest behavior shifts may occur early post-diagnosis, with subsequent trajectories either stabilizing or partially reverting over time, and that initial post-diagnosis changes in behavior may not be sustained over time. This finding aligns with existing literature on “teachable moments” in cancer care, highlighting that early survivorship may be a limited window in which survivors are most receptive to lifestyle change.^30-31^ The adoption of healthier behaviors during and soon after cancer treatment may not be sustained in the long term as initial motivations wane, routine medical oversight lessens, and long-term coping patterns may change.^32^ This underscores the importance of addressing alcohol use as a dynamic, long-term behavior, and the need for repeated counseling throughout survivorship with regular reassessment and coordinated care between oncology and primary care providers to ensure sustained lifestyle improvements and better long-term health outcomes.

Strengths of this study include its longitudinal design, a large sample size, comprehensive collection of clinical, demographic, and lifestyle data, and its focus on a relatively unexplored phase of survivorship. To our knowledge, this is among the first studies to explore predictors of alcohol intake in BC survivors several years after diagnosis, providing insight into long-term behavioral patterns and their determinants. While our findings have important implications, they must be interpreted considering potential limitations. Supplemental analyses indicate that patients who were White, premenopausal, had lower-stage disease, did not receive chemotherapy or radiation, and reported higher physical and mental health scores were more likely to complete the Year 4 survey. These differences suggest that our sample may underrepresent survivors with more intensive treatment histories or poorer health status. The study is limited by lack of detailed information on the type of alcohol consumed (such as wine, beer, or spirits) and the context of consumption (e.g., with meals or binge drinking). Additionally, alcohol intake was self-reported and subject to recall and social desirability bias, which may have led to underreporting. Our sample was also demographically homogeneous with the vast majority being White and well-educated, limiting generalizability to more diverse populations.

In conclusion, many BC survivors drink alcohol four years after a BC diagnosis (a greater proportion than we found previously at one year after a BC diagnosis).^19^ These findings highlight the importance of continued, nuanced health behavior counseling in both early and late survivorship care, particularly targeting younger survivors, smokers, and those with more active lifestyles (perhaps because they may not perceive their alcohol intake as a health risk). Further research is warranted to explore the motivations underlying changes in alcohol use during cancer survivorship and to develop tailored interventions to support long-term health in this population.

## Figures and Tables

**Table 1:** Characteristics of survey respondents and univariable analyses of Year 4 alcohol intake by characteristics

	Alcoholic drinks per week at year 4					
	None (N=217)	1-4 (N=351)	5-14 (N=134)	15+ (N=17)	Total (N=719)	P- value^1^
**Gender,** n (%)						0.138
Female	214 (30.1%)	350 (49.2%)	131 (18.4%)	17 (2.4%)	712 (99.0%)	
Male	3 (42.9%)	1 (14.3%)	3 (42.9%)	0 (0.0%)	7 (1.0%)	

**Age at cancer diagnosis,** n (%)						<0.001
< 40	3 (6.4%)	33 (70.2%)	8 (17.0%)	3 (6.4%)	47 (6.5%)	
40-49	33 (26.4%)	67 (53.6%)	20 (16.0%)	5 (4.0%)	125 (17.4%)	
50-59	59 (28.9%)	99 (48.5%)	41 (20.1%)	5 (2.5%)	204 (28.4%)	
60-69	67 (33.5%)	94 (47.0%)	37 (18.5%)	2 (1.0%)	200 (27.8%)	
70+	55 (38.5%)	58 (40.6%)	28 (19.6%)	2 (1.4%)	143 (19.9%)	

**Race**, n (%)						0.306
White	208 (29.8%)	342 (49.0%)	132 (18.9%)	16 (2.3%)	698 (97.1%)	
Non-White/not disclosed	9 (42.9%)	9 (42.9%)	2 (9.5%)	1 (4.8%)	21 (2.9%)	

**Education**, n (%)						0.794
High school graduate or less	30 (36.1%)	33 (39.8%)	18 (21.7%)	2 (2.4%)	83 (11.6%)	
Vocational education beyond high school	21 (31.8%)	32 (48.5%)	12 (18.2%)	1 (1.5%)	66 (9.2%)	
Some college or associate’s degree	57 (33.5%)	75 (44.1%)	33 (19.4%)	5 (2.9%)	170 (23.7%)	
Bachelor's degree	52 (26.8%)	102 (52.6%)	35 (18.0%)	5 (2.6%)	194 (27.1%)	
Graduate school	55 (27.1%)	108 (53.2%)	36 (17.7%)	4 (2.0%)	203 (28.4%)	
Missing	2	1	0	0	3	

**Household finance situation**, n (%)						0.947
Difficulty paying bills or little spare money for extra things	55 (30.9%)	85 (47.8%)	33 (18.5%)	5 (2.8%)	178 (25.0%)	
After paying bills, still enough money for extra things	161 (30.1%)	263 (49.3%)	98 (18.4%)	12 (2.2%)	534 (75.0%)	
Missing	1	3	3	0	7	

**Menopausal status,** n (%)						0.464
Pre-menopausal	47 (27.8%)	83 (49.1%)	33 (19.5%)	6 (3.6%)	169 (25.9%)	
Post-menopausal	155 (32.1%)	230 (47.6%)	89 (18.4%)	9 (1.9%)	483 (74.1%)	
Missing	15	38	12	2	67	

**Smoking status at baseline,** n (%)						<0.001
Never	163 (34.2%)	245 (51.4%)	62 (13.0%)	7 (1.5%)	477 (67.2%)	
Former	48 (22.0%)	95 (43.6%)	67 (30.7%)	8 (3.7%)	218 (30.7%)	
Current	4 (26.7%)	6 (40.0%)	3 (20.0%)	2 (13.3%)	15 (2.1%)	
Missing	2	5	2	0	9	

**Minutes of strenuous exercise per week at baseline,** n (%)						0.1562
0	136 (32.2%)	202 (47.9%)	71 (16.8%)	13 (3.1%)	422 (69.3%)	
1-59	12 (31.6%)	18 (47.4%)	8 (21.1%)	0 (0.0%)	38 (6.2%)	
60-119	14 (23.3%)	32 (53.3%)	14 (23.3%)	0 (0.0%)	60 (9.9%)	
120+	16 (18.0%)	48 (53.9%)	22 (24.7%)	3 (3.4%)	89 (14.6%)	
Missing	39	51	19	1	110	

**Minutes of moderate exercise per week at baseline**, n (%)						0.001
0	70 (36.3%)	92 (47.7%)	29 (15.0%)	2 (1.0%)	193 (30.0%)	
1-119	42 (24.7%)	94 (55.3%)	34 (20.0%)	0 (0.0%)	170 (26.4%)	
120-239	42 (25.0%)	78 (46.4%)	41 (24.4%)	7 (4.2%)	168 (26.1%)	
240+	33 (29.5%)	53 (47.3%)	18 (16.1%)	8 (7.1%)	112 (17.4%)	
Missing	30	34	12	0	76	

**Minutes of mild exercise per week at baseline**, n (%)						0.005
0	49 (35.5%)	62 (44.9%)	23 (16.7%)	4 (2.9%)	138 (20.7%)	
1-119	49 (23.7%)	121 (58.5%)	34 (16.4%)	3 (1.4%)	207 (31.0%)	
120-239	57 (30.5%)	96 (51.3%)	31 (16.6%)	3 (1.6%)	187 (28.0%)	
240+	39 (28.9%)	52 (38.5%)	37 (27.4%)	7 (5.2%)	135 (20.2%)	
Missing	23	20	9	0	52	

**Tumor stage**, n (%)						0.477
0	36 (28.6%)	67 (53.2%)	22 (17.5%)	1 (0.8%)	126 (17.6%)	
1	113 (31.9%)	167 (47.2%)	68 (19.2%)	6 (1.7%)	354 (49.4%)	
2	56 (28.0%)	101 (50.5%)	34 (17.0%)	9 (4.5%)	200 (27.9%)	
3	11 (29.7%)	16 (43.2%)	9 (24.3%)	1 (2.7%)	37 (5.2%)	
Missing	1	0	1	0	2	

**Type of surgery**, n (%)						0.548
Lumpectomy only	109 (31.0%)	174 (49.4%)	63 (17.9%)	6 (1.7%)	352 (51.3%)	
Mastectomy	96 (28.7%)	160 (47.9%)	68 (20.4%)	10 (3.0%)	334 (48.7%)	
Missing	12	17	3	1	33	

**Patient underwent endocrine therapy,** n (%)						0.809
No	78 (31.5%)	123 (49.6%)	42 (16.9%)	5 (2.0%)	248 (34.5%)	
Yes	139 (29.5%)	228 (48.4%)	92 (19.5%)	12 (2.5%)	471 (65.5%)	

**Patient underwent chemotherapy**, n (%)						0.551
No	154 (29.4%)	255 (48.8%)	103 (19.7%)	11 (2.1%)	523 (72.7%)	
Yes	63 (32.1%)	96 (49.0%)	31 (15.8%)	6 (3.1%)	196 (27.3%)	

**Patient underwent radiotherapy**, n (%)						0.123
No	98 (28.6%)	160 (46.6%)	75 (21.9%)	10 (2.9%)	343 (47.7%)	
Yes	119 (31.6%)	191 (50.8%)	59 (15.7%)	7 (1.9%)	376 (52.3%)	

**Pathologic BRCA1/2 mutation**, n (%)						0.077
No	216 (30.7%)	341 (48.5%)	130 (18.5%)	16 (2.3%)	703 (97.8%)	
Yes	1 (6.3%)	10 (62.5%)	4 (25.0%)	1 (6.3%)	16 (2.2%)	

**ER status of first BC**, n (%)						0.395
Negative	28 (26.9%)	59 (56.7%)	16 (15.4%)	1 (1.0%)	104 (14.6%)	
Positive	188 (31.0%)	288 (47.5%)	115 (19.0%)	15 (2.5%)	606 (85.4%)	
Missing	1	4	3	1	9	

**PR status of first BC**, n (%)						0.343
Negative	54 (33.8%)	81 (50.6%)	23 (14.4%)	2 (1.3%)	160 (22.6%)	
Positive	162 (29.6%)	264 (48.3%)	108 (19.7%)	13 (2.4%)	547 (77.4%)	
Missing	1	6	3	2	12	

**HER2 status of first BC**, n (%)						0.096
Negative	154 (31.4%)	235 (47.9%)	92 (18.7%)	10 (2.0%)	491 (87.7%)	
Positive	19 (27.5%)	35 (50.7%)	10 (14.5%)	5 (7.2%)	69 (12.3%)	
Missing	44	81	32	2	159	

**Categorized PROMIS global mental health T-score, baseline**, n (%)						0.063
< 46	317 (66.2%)	122 (25.5%)	37 (7.7%)	3 (0.6%)	479 (23.1%)	
46-52	307 (60.9%)	148 (29.4%)	43 (8.5%)	6 (1.2%)	504 (24.3%)	
53-55	219 (60.5%)	115 (31.8%)	26 (7.2%)	2 (0.6%)	362 (17.5%)	
56+	406 (55.7%)	243 (33.3%)	73 (10.0%)	7 (1.0%)	729 (35.1%)	
Missing	3	2	1	0	6	

**PROMIS global physical health T-score, baseline**, n (%)						<.0.01
< 46	353 (70.7%)	110 (22.0%)	34 (6.8%)	2 (0.4%)	499 (24.4%)	
46-52	368 (60.8%)	186 (30.7%)	44 (7.3%)	7 (1.2%)	605 (29.5%)	
53-55	216 (54.3%)	141 (35.4%)	40 (10.1%)	1 (0.3%)	398 (19.4%)	
56+	295 (54.0%)	186 (34.1%)	57 (10.4%)	8 (1.5%)	546 (26.7%)	
Missing	20	7	5	0	32	

1. Fisher exact test

**Table 2.** Comparison of alcohol intake per week as reported in the baseline survey to that reported in the Year 4 survey

Drinks per week, baseline	Drinks per week, Year 4				
	None	1-4	5-14	15+	Total
None	170	28	1	0	199 (27.7%)
1-4	42	268	36	1	347 (48.3%)
5-14	3	54	87	7	151 (21.0%)
15+	2	1	10	9	22 (3.1%)
Total	217 (30.2%)	351 (48.8%)	134 (18.6%)	17 (2.4%)	719

**Table 3:** Univariable associations of change in alcohol use from baseline to year 4 with demographic and clinical characteristics

	Change in alcohol use from baseline to year 4				
	Decrease from baseline to year 4 (N=99)	No change from baseline to year 4 (N=355)	Increase from baseline to year 4 (N=44)	Total (N=498)	P- value^1^
**Gender,** n (%)					0.164
Female	97 (19.7%)	353 (71.6%)	43 (8.7%)	493 (99.0%)	
Male	2 (40.0%)	2 (40.0%)	1 (20.0%)	5 (1.0%)	

**Age at cancer diagnosis,** n (%)					0.459
< 40	7 (17.1%)	28 (68.3%)	6 (14.6%)	41 (8.2%)	
40-49	19 (20.9%)	60 (65.9%)	12 (13.2%)	91 (18.3%)	
50-59	24 (16.9%)	106 (74.6%)	12 (8.5%)	142 (28.5%)	
60-69	29 (21.0%)	99 (71.7%)	10 (7.2%)	138 (27.7%)	
70+	20 (23.3%)	62 (72.1%)	4 (4.7%)	86 (17.3%)	

**Race,** n (%)					0.331
White	95 (19.5%)	348 (71.5%)	44 (9.0%)	487 (97.8%)	
Non-White/not disclosed	4 (36.4%)	7 (63.6%)	0 (0.0%)	11 (2.2%)	

**Education**, n (%)					0.366
High school graduate or less	10 (18.9%)	41 (77.4%)	2 (3.8%)	53 (10.7%)	
Vocational education beyond high school	11 (25.0%)	28 (63.6%)	5 (11.4%)	44 (8.9%)	
Some college or associate’s degree	30 (25.9%)	77 (66.4%)	9 (7.8%)	116 (23.3%)	
Bachelor's degree	27 (19.3%)	100 (71.4%)	13 (9.3%)	140 (28.2%)	
Graduate school	21 (14.6%)	108 (75.0%)	15 (10.4%)	144 (29.0%)	
Missing	0	1	0	1	

**Household finance situation,** n (%)					0.095
Difficulty paying bills or little spare money for extra things	28 (22.6%)	80 (64.5%)	16 (12.9%)	124 (25.2%)	
After paying bills, still enough money for extra things	71 (19.2%)	271 (73.4%)	27 (7.3%)	369 (74.8%)	
Missing	0	4	1	5	

**menopausal status**, n (%)					0.084
Pre-menopausal	23 (19.3%)	79 (66.4%)	17 (14.3%)	119 (26.5%)	
Post-menopausal	67 (20.3%)	239 (72.4%)	24 (7.3%)	330 (73.5%)	
Missing	9	37	3	49	

**Smoking status at baseline,** n (%)					0.175
Never	62 (19.6%)	233 (73.5%)	22 (6.9%)	317 (64.8%)	
Former	32 (19.6%)	112 (68.7%)	19 (11.7%)	163 (33.3%)	
Current	2 (22.2%)	5 (55.6%)	2 (22.2%)	9 (1.8%)	
Missing	3	5	1	9	

**Minutes of strenuous exercise per week at baseline,** n (%)					0.011
0	59 (21.0%)	201 (71.5%)	21 (7.5%)	281 (66.1%)	
1-59	10 (34.5%)	17 (58.6%)	2 (6.9%)	29 (6.8%)	
60-119	9 (19.6%)	31 (67.4%)	6 (13.0%)	46 (10.8%)	
120+	5 (7.2%)	53 (76.8%)	11 (15.9%)	69 (16.2%)	
Missing	16	53	4	73	

**Minutes of moderate exercise per week at baseline**, n (%)					0.077
0	33 (26.2%)	85 (67.5%)	8 (6.3%)	126 (27.9%)	
1-119	22 (17.6%)	96 (76.8%)	7 (5.6%)	125 (27.7%)	
120-239	21 (16.7%)	91 (72.2%)	14 (11.1%)	126 (27.9%)	
240+	13 (17.3%)	50 (66.7%)	12 (16.0%)	75 (16.6%)	
Missing	10	33	3	46	

**Minutes of mild exercise per week at baseline**, n (%)					0.012
0	18 (19.4%)	66 (71.0%)	9 (9.7%)	93 (19.9%)	
1-119	24 (15.3%)	122 (77.7%)	11 (7.0%)	157 (33.6%)	
120-239	30 (23.1%)	94 (72.3%)	6 (4.6%)	130 (27.8%)	
240+	20 (23.0%)	51 (58.6%)	16 (18.4%)	87 (18.6%)	
Missing	7	22	2	31	

**Tumor stage**, n (%)					0.743
0	20 (23.5%)	58 (68.2%)	7 (8.2%)	85 (17.1%)	
1	49 (19.8%)	179 (72.2%)	20 (8.1%)	248 (49.9%)	
2	25 (17.9%)	103 (73.6%)	12 (8.6%)	140 (28.2%)	
3	5 (20.8%)	15 (62.5%)	4 (16.7%)	24 (4.8%)	
Missing	0	0	1	1	

**Type of surgery**, n (%)					0.142
Lumpectomy only	53 (21.2%)	181 (72.4%)	16 (6.4%)	250 (52.3%)	
Mastectomy	39 (17.1%)	164 (71.9%)	25 (11.0%)	228 (47.7%)	
Missing	7	10	3	20	

**Patient underwent endocrine therapy,** n (%)					0.047
No	45 (25.6%)	119 (67.6%)	12 (6.8%)	176 (35.3%)	
Yes	54 (16.8%)	236 (73.3%)	32 (9.9%)	322 (64.7%)	
**Patient underwent chemotherapy**, n (%)					0.848
No	69 (19.2%)	258 (71.9%)	32 (8.9%)	359 (72.1%)	
Yes	30 (21.6%)	97 (69.8%)	12 (8.6%)	139 (27.9%)	

**Patient underwent radiotherapy,** n (%)					0.080
No	45 (19.0%)	164 (69.2%)	28 (11.8%)	237 (47.6%)	
Yes	54 (20.7%)	191 (73.2%)	16 (6.1%)	261 (52.4%)	

**Pathologic BRCA1/2 mutation**, n (%)					0.685
No	97 (20.1%)	344 (71.2%)	42 (8.7%)	483 (97.0%)	
Yes	2 (13.3%)	11 (73.3%)	2 (13.3%)	15 (3.0%)	

**ER status of first BC**, n (%)					0.442
Negative	20 (23.8%)	59 (70.2%)	5 (6.0%)	84 (17.1%)	
Positive	77 (18.9%)	293 (71.8%)	38 (9.3%)	408 (82.9%)	
Missing	2	3	1	6	

**PR status of first BC**, n (%)					0.252
Negative	28 (23.7%)	83 (70.3%)	7 (5.9%)	118 (24.1%)	
Positive	68 (18.3%)	268 (72.0%)	36 (9.7%)	372 (75.9%)	
Missing	3	4	1	8	

**HER2 status of first BC**, n (%)					0.096
Negative	61 (17.9%)	253 (74.2%)	27 (7.9%)	341 (88.6%)	
Positive	12 (27.3%)	26 (59.1%)	6 (13.6%)	44 (11.4%)	
Missing	26	76	11	113	


**Categorized PROMIS global mental health T score, baseline**, n (%)					0.290
< 46	23 (28.0%)	51 (62.2%)	8 (9.8%)	82 (16.6%)	
46-52	20 (15.0%)	104 (78.2%)	9 (6.8%)	133 (26.9%)	
53-55	21 (21.4%)	68 (69.4%)	9 (9.2%)	98 (19.8%)	
56+	35 (19.2%)	129 (70.9%)	18 (9.9%)	182 (36.8%)	
Missing	0	3	0	3	

**PROMIS global physical health T score, baseline,** n (%)					0.448
< 46	23 (26.7%)	56 (65.1%)	7 (8.1%)	86 (17.4%)	
46-52	26 (18.3%)	104 (73.2%)	12 (8.5%)	142 (28.8%)	
53-55	22 (18.3%)	91 (75.8%)	7 (5.8%)	120 (24.3%)	
56+	28 (19.3%)	100 (69.0%)	17 (11.7%)	145 (29.4%)	
Missing	0	4	1	5	

1. Fisher exact test

## Data Availability

The datasets generated during and/or analyzed during the current study are not publicly available due to institution confidentiality purposes but are available from the corresponding author on reasonable request.
